# Abstracts

**DOI:** 10.1155/S0962935194000761

**Published:** 1994


Mediators of Inflammation 3, S49–S51 (1994)

### Abstracts

### Inhaled cromoglycate versus nedocromil: clinical results

*L. Armenio, L. Brunetti, D. Colazzo and F. Cardinale*
Istituto di Pediatria Clinica e Sociale, Università di Bari, Policlinico, Piazza G. Cesare, I-70124 Bari, Italy

Recent national and international protocols recommend the use of anti-inflammatory agents for the long-term treatment of asthma both in children and in adults. Because of their biologically demonstrated anti-inflammatory properties, and their efficacy and safety in the clinical setting, sodium cromoglycate and nedocromil sodium are among the first-line drugs used as alternatives to, or in association with, topically applied steroids. Although some experimental and clinical evidence seems to indicate that nedocromil is more effective than sodium cromoglycate in treating asthma, the issue is still open. In an attempt to contribute to the debate, the results of clinical studies that have been performed with these two cromones have been reviewed.

### Reduced priming capacity of bronchoalveolar lavage liquid on polymorphonuclear leukocytes after nedocromil sodium therapy in asthmatic children

*R. Gabbianelli,*[1] *A. Kantar,*[2] *N. Oggiano,*[2] *M. Gentili,*[3] *R. Pagni,*[3] *G. Falcioni*[1] *and P. L. Giorgi*[2]

[1]Department of Biology M.C.A., University of Camerino, Via Camerini, 2, 62032 Camerino, Italy; [2]Department of Paediatrics, University of Ancona, Ancona, Italy; [3]Intensive Care Unit, Children's Hospital, Ancona, Italy

A variety of inflammatory cell types and mediators of inflammation have been noted in the broncoalveolar lavage (BAL) of asthmatic patients. These components exhibit a priming on phagocytic cells. The priming effect of BAL on polymorphonuclear leukocytes (PMNs) was studied in asthmatic children before and after nedocromil therapy, using a chemiluminescence technique. Our data demonstrate a significant reduction in the priming activity of BAL after nedocromil sodium therapy. This effect can be partially attributed to the action of nedocromil on inflammatory cells and on the release of mediators.

### Anti-inflammatory therapy with nedocromil in chronic bronchopneumopathy (asthma and cystic fibrosis)

*G. Caramia, F. Franceschini and R. Gagliardini*
Paediatric-Neonatology Division, G. Salesi Hospital, Via Corridoni 11, I-60123 Ancona, Italy

Preliminary results of two parallel and complementary studies whose aim is to evaluate the efficacy of long-term therapy with inhaled nedocromil (NC) in patients affected with mild-moderate asthma and with cystic fibrosis (CF) are reported. Before and after 2 months of therapy with NC, 16 patients (ten with asthma and six with CF) were examined and evaluated through clinical data, fundamental spirometric parameters (FVC, FEV1, FEF25-75), and bronchial hyper-reactivity (BHR). The results obtained confirm the efficacy of NC in improving spirometric data and BHR in asthmatic patients. Using the same parameters, the therapeutic benefits of NC in children with CF appeared less evident, this is probably due to the numerous factors that can influence bronchial inflammation in CF.

### Effect of inhaled nedocromil sodium therapy on blood gas changes in asthmatic children challenged with ultrasonically nebulized distilled water: a comparison of 8 mg versus 16 mg daily dose

*N. Oggiano, A. Kantar, S. Bruni, F. M. Cutrona, E. Fabbrizi, R. Piccinini and P. L. Giorgi*
Department of Paediatrics, University of Ancona, Via Corridoni 11, I-60123 Ancona, Italy

Changes in the time courses of blood partial pressures of oxygen ($Pto_2$) and carbon dioxide ($Ptco_2$) before, during and after challenge with ultrasonically nebulized distilled water (UNDW) were evaluated in 22 children with mild asthma in basal conditions, and after 8 weeks of therapy with inhaled nedocromil sodium at a daily dosage of 8 or 16 mg. $Pto_2$ and $Ptco_2$ were followed, using a transcutaneous $o_2$ and $co_2$ monitoring system. All asthmatic subjects presented a significant decrease in $Pto_2$ and/or $Ptco_2$ (>20% basal value) during or after challenge. After therapy, the decrease in $Pto_2$ and $Ptco_2$ was normalized in the group treated with 16 mg/day, whereas only a partial yet significant reduction in the decrease of $o_2$ and $co_2$ was observed in the group assuming 8 mg/day. These data

© 1994 Rapid Communications of Oxford Ltd indicate that inhaled nedocromil is effective in treating bronchial hyper-responsiveness in childhood, and that the dose required to achieve this effect is 16 mg/day.

### The effects of SCG and ipratropium bromide on two bronchial challenges (hyperosmolar solution and exercise)

*M. Miraglia del Giudice, Jr, F. Decimo, C. Capristo, L. Masini and N. Maiello*
I and IV Paediatric Clinic, II University of Naples, Naples, Italy

To understand how exercise induced asthma (EIA) may be related to changes in airway osmolarity, a study was made of the inhibitory effects of two drugs, ipratropium bromide and SCG on two bronchial challenges; namely hyperosmolar solution (HY) and exercise (EX), in 15 asthmatic children. The children were screened by EX and HY tests at the beginning and after pretreatment (double-blind) with either placebo, or 10 mg SCG, or 80 mg ipratropium bromide. Both drugs were able to inhibit the challenge with HY and EX in almost all the patients. However, because the protection against HY was more significant than against EX, our data suggest that hyperosmolarity may not be the only mechanism involved in EIA.

### Clinical-epidemiological evaluation of the use of disodiumcromoglycate from 1980 to 1992

*G. L. Grzincich, G. Pisi, M. E. Street, A. Pelizzoni and A. Battistini*
Centro di Fisiopatologia Respiratoria Infantile, and Clinica Pediatrica, Università di Parma Via Gramsci 14, 43100, Parma, Italy

The examination of data from 1925 asthmatic patients (9 months–18 years) pointed to a significant increase in the use of beta-2-stimulators, ketotifen and nedocromil sodium from 1980 to 1992; on the other hand, the use of disodiumcromoglycate (DSCG) and of theophilline was unchanged. General physicians prescribe DSCG principally in allergic patients, or if they have made a diagnosis of asthma. Of the three formulations of DSCG, phials are used in the younger children, capsules and spray in the older ones and in allergic subjects. DSCG in our study was taken correctly in 57% of the cases.

### Asthma and sport: efficacy of a kind of sport or a kind of therapy?

*M. Canciani*
Children's Hospital 'Burlo Garofolo', Via dell'Istria, 65/1, I-34137 Trieste, Italy

The study was carried out on 17 mildly atopic asthmatics (mean age 14 ± 5.3 years; $FEV_1$ pred. 87–108%) performing sport for more than 3 years and for at least 6 months each year. Ten patients were swimmers and seven footballers. Clinical data and details of drug use were compared at the beginning and at the end of the observation period (at the conclusion of which a bronchial provocation test with methacoline was also carried out). Clinical and drug-use scores, comparable at the beginning of the physical exercise in both groups, showed a statistical difference at the end of the study in the swimming group ($p < 0.05$). Only one swimmer (10%) had a positive methacoline result, compared with three footballers (43%). Comparing the drug-related scores, statistical significance only resulted for sodium cromoglycate ($p < 0.05$). In the absence of a pure study comparing two drug-free populations, it is difficult to say whether improvement in the swimmers is due to this sport or to the effects of cromoglycate.

### An assessment of bronchial hyper-reactivity in children with atopic rhinitis

*S. La Grutta,[1] G. Cuttitta,[2] F. Cibella,[3] G. Panascì[1] and S. Spedale[1]*

[1]Department of Paediatrics, Ospedale 'G. di Cristina', Piazza Montalto, 4-90134, Palermo, Italy; [2]Istituto di Scienza e Tecnologia dello Sport e dell'Attività Fisica, and [3]Istituto di Fisiopatologia Respiratoria, C.N.R., Palermo, Italy

Aspecific bronchial hyper-reactivity is frequently found in atopic children. This study involved 41 children affected by atopic rhinitis with normal pulmonary function and without any symptoms relevant to bronchial asthma in their personal histories. All the subjects were submitted to the methacholine bronchial provocation test for the evaluation of bronchial hyper-reactivity (BHR). Twenty-three subjects (56%) demonstrated a mean methacholine dose, producing a 20% $FEV_1$ reduction ($PD_{20}$) of 792 mcg ( ± 535 SD) and were labelled responders (R), while the remaining 18 were non-responders (NR). A significant difference was found between R and NR as regards the age of atopic symptom onset ($p = 0.037$). A close positive linear relationship between $PD_{20}$ and subject age was found ($p = 0.0099$). Fourteen of the 23 R were admitted to an 8-week treatment with inhaled nedocromil sodium (NS) 4 mg, 4 times a day. At the end of the treatment, a second bronchial challenge was performed: the $PD_{20}$ appeared to be significantly reduced (45%, $p = 0.034$). It is concluded that children with atopic rhinitis demonstrate a high prevalence of BHR. Nedocromil sodium, because of its effectiveness, has to be evaluated as first choice therapy in the treatment of BHR in absence of airway obstruction.

### Preventive effect of nedocromil sodium versus sodium cromoglycate on exercise-induced bronchoconstriction in children

*C. Caffarelli,[1] G. Cavagni,[2] I. Stapane[1] and C. Rossi[1]*

[1]Clinica Pediatrica, Università di Parma, Via Gramsci, 14, 43100 Parma, Italy; [2]Clinica Pediatrica, Università di Brescia, Brescia, Italy

A comparison was made between the protective effects of nedocromil sodium (NS) and sodium cromoglycate (SCG) on exercise-induced asthma (EIA), in a double-blind cross-over study in asthmatic children. Patients performed 6 min of exercise without any treatment. Then, the same patients performed 6 min of exercise 20 min after administration of 4 mg of NS and 20 mg of SCG. Differences between spirometric values (maximum percentage PEF fall and $FEV_1$ fall at 15 min) were not statistically significant for comparisons between the two active treatments. Nedocromil sodium was found to be as effective as SCG in preventing EIA.

### Effectiveness of nedocromil sodium in preventing exercise-induced asthma in children

*M. Verini, A. Verrotti, M. P. Del Duca and G. Morgese*
Department of Pediatrics, University of Chieti, Ospedale Pediatrico, Via Nicolini 11, 66100 Chieti, Italy

Exercise-induced asthma is a well-known phenomenon, which particularly affects children and which has an important social impact. In order to assess the usefulness of nedocromil sodium in the prevention of exercise-induced asthma, 49 (15 female, 34 male) children who suffered from asthma were studied; their mean ± SD age was 9.2 ± 3.0 (range: 3.3–19.1) years. On the first day, respiratory function was evaluated by spirometry, basally and after a 6 min run. The inhalation of nedocromil sodium had a great influence on post-exercise lung function measurements; in fact, on the day of nedocromil sodium pretreatment, our patients showed an increase of respiratory function which was significantly different from the parameters recorded during the first day. Our findings suggest that nedocromil sodium is effective in the prevention of exercise-induced asthma in paediatric age.

### Problems of lung inflammation in cystic fibrosis

*G. Cazzola*
Cystic Fibrosis Center, Verona, Italy

The progressive pulmonary disease found in cystic fibrosis (CF) which is associated with *Pseudomonas aeruginosa* infection has been related to the chronic severe inflammatory host response. Thus, therapy that reduce pulmonary inflammation may prove to have clinical efficacy. A wide variety of agents that may modulate this inflammatory vicious circle are being investigated and promising results are coming out. Furthermore, large controlled trials are still necessary to confirm clinical efficacy and to continue improvement in the clinical course of CF patients.

### The long-term effects of nedocromil sodium on bronchial responsiveness to methacholine and respiratory symptoms in atopic asthmatic children

*M. Pifferi, M. Baldini, C. Bertelloni, S. Cecchetti, G. Fruzza and G. Baldini*
Lung Function Laboratory and Allergy Center, Paediatric Clinic, University of Pisa, Via Roma 35, 56100 Pisa, Italy

A long-term study of the effect of nedocromil sodium (NS) on lung function, bronchial responsiveness to methacholine, respiratory symptoms, corticosteroid and/or bronchodilator intake in 15 atopic asthmatic children is described. The study was divided into a baseline period (8 weeks), a treatment period (16 weeks), and a wash-out period (8 weeks). Two inhalations of NS (2 mg per actuation) were administered three times daily by metered dose pressurised aerosol. Values of FEV1 did not change significantly, but increased slightly after 8 and 16 weeks of treatment with NS. Significant changes ($p = 0.001$) in FEF 25-75 with regard to the pretreatment values were recorded at the end of the treatment period. PD20FEV1 increased significantly after 8 ($p < 0.001$) and 16 weeks ($p < 0.001$). Asthma symptoms, and corticosteroid or bronchodilator intake, decreased significantly after 8 and 16 weeks of treatment. In conclusion, NS appears to be both effective and safe in the long-term treatment of asthma in children.

